# Supplementary material for: Megacystis in the first trimester of pregnancy: Prognostic factors and perinatal outcomes
Source: PLoS One. 2021 Sep 7;16(9):e0255890. doi: 10.1371/journal.pone.0255890 (PMC8423287; doi:10.1371/journal.pone.0255890)
Supplement: Attachment — Submitted filename: Response to reviewer.docx [file pone.0255890.s002.docx]

**Emmanuelle LESIEUR, MD**

Department of Gynaecology and Obstetrics,

Gynépole, Hôpital Nord

AMU, Aix- Marseille University

AP-HM

France Adress : Chemin des Bourrely

13015, Marseille, France

Phone Number : +33 4 91 96 48 53

Mail : [emmanuelle.lesieur@ap-hm.fr](mailto:emmanuelle.lesieur@ap-hm.fr) ;

To **Plos One Editorial**

The 18^rd^ of March 2021

Thank you very much for reviewing our manuscript “***Megacystis in the first trimester of pregnancy: prognostic factors and perinatal outcomes***”. we also greatly appreciate the reviewers for their complimentary comments and suggestions. we have carried out the experiments that the reviewers suggested and we revised the manuscript accordingly.

Here is our response to comments :

I. **Journal Requirements**
1. **Please ensure that your manuscript meets PLOS ONE's style requirements, including those for file naming.**

Changes have been made. Our manuscript meet now Plos One’s style requirements.

2. **You indicated that you had ethical approval for your study.
   In your Methods section, please ensure you have also stated whether you obtained consent from parents or guardians of the minors included in the study (if applicable) or whether the research ethics committee or IRB specifically waived the need for their consent.**

The details were made at the beginning of the material and methods paragraph.

*L. 93. All patients were informed during ultrasound examinations of the possible use of their data for scientific purposes (written informed consent given for adults, minors and people under guardianship), and could any time indicate their refusal. A request for processing of this data was made to the Committee of Patient Data Protection and was accepted.*

**3. In the ethics statement in the manuscript and in the online submission form, please provide additional information about the patient records/samples used in your retrospective study.
Specifically, please ensure that you have discussed whether all data/samples were fully anonymized before you accessed them and/or whether the IRB or ethics committee waived the requirement for informed consent.
If patients provided informed written consent to have data/samples from their medical records used in research, please include this information.**

Patients provided informed written consent to have data from their medical records used in research.

At the beginning of the medical consultation, all couple receive a manuscript, in French, in which they consent that explorations being used for research or publication. Here is the document that the couple signs.

Then, we have to declare the studies to the “PADS”. This is an ethics committee which ensures data protection and respect for rights in accordance with the European General Data Protection Regulations and applicable French Law; They ensure that all data are fully anonymized.

Details have been made in the manuscript *(L. 94-95)*

II. **Additional Editor Comments**
1. **The authors included a large series of first trimester fetuses with megacystis and described the neonatal outcomes according to the bladder size.
   I agree with one of the reviewers that this large series merits publication on this Journal but I also agree with the main criticism by the second reviewer that bladder size groups were arbitrarily selected and that fetal bladder size should be consider a continuous variable and therefore I strongly recommend to assess the clinical value of bladder size to predict neonatal outcome by a decision tree analysis, which allow an automatic classification using the predictive variable as a continuous variable allowing a sequential analysis to predict postnatal outcome. Since previous studies have demonstrated that the size of fetal bladder predicts adverse neonatal outcome, I think that assessing an automatic fetal bladder size cut-off may be of clinical interest and thus, deserving to be publish in this Journal**

We thank the reviewer for this positive statement. We reanalyzed the cohort by considering the bladder size as a continuous variable (developed in the next responses to comments).

2. **Please include the following references:
   Fontanella et al. Antenatal Workup of Early Megacystis and Selection of Candidates for Fetal Therapy. Fetal Diagn Ther 2019;45(3):155-161.**

This reference has been included :

*L 367.*

*27.	Fontanella F, Duin L, Adama van Scheltema PN, Cohen-Overbeek TE, Pajkrt E, Bekker M, et al. Antenatal Workup of Early Megacystis and Selection of Candidates for Fetal Therapy. Fetal Diagn Ther. 2019;45(3):155–61.*

III. **Comments to the Author**

**Reviewer #1: The authors report a multicenter cohort study, which evaluates the prognosis associated with the bladder size at 1st trimester in case of megacystis.
This large and interesting series deserve to be published. However it should be more focus on its originality that lies in the analysis of outcomes based on bladder size. Moreover, data on evolution during pregnancies and renal function should be provided.**

We thank the reviewer for this positive statement.

We reanalyzed the cohort by considering the bladder size as a continuous variable

With this analysis, we can consider two groups : One with favorable outcomes, with or without urological problems (n = 12) and one with adverse outcome (n = 63)

The average bladder size for each group is as follows : 10,2 mm for favorable outcome with a standard deviation of 2,4 and 23,2 mm for adverse outcome with a standard deviation of 10,8, p < 0,001 (Mann-Whitney test).

This analysis was done with this two groups to consider the final response : favorable or adverse outcome.

Analysis with three groups ( bladder size 7-10 mm, 11 – 15, > 15 mm) was justified with references. In the original article of Kao C. *et al* : *Perinatal outcome and prognostic factors of fetal megacystis diagnosed at 11-14 week’s gestation, Prenat Diagn, 2020 Nov 21. Doi:10.1002/pd.5868*, with a total of 98 cases of first trimester megacystis, authors have shown that :

- Longitudinal bladder diameter > 15 mm is highly associated with obstructive uropathy
- Isolated megacystis with longitudinal bladder diameter < 12 mm was associated with good outcome and no cases of obstructive uropathy.

This conclusion is in line with ours. If bladder size is a continuous variable, there is a favorable outcome when the bladder size is about 10.2 mm with DS 2 mm.

These results were reported in the manuscript :

*L 152 – L 157 : Fetal outcome according to bladder size*

*Two groups were considered : one with favorable outcome, with or without urological problems (n = 12) and one with adverse outcome (n=63). In this analysis, bladder size was considered as a continuous variable. The average bladder size for each group is as follows : 10,2 mm for favorable outcome with a standard deviation of 2,4 and 23,2 mm for adverse outcome with a standard deviation of 10,8, p < 0,001.*

Regarding the assessment of renal function, we thank this reviewer for this comment because this remark is relevant and appropriate. it is not part of current practice in the two university hospitals where the study was carried out. However, we know that it is a major prognostic factor in cases of megacystis. This is therefore one of the limits of our work. We added this limit in the discussion and add references.

*L 242 – 244 : Finally, although it is known as a prognostic factor (20,37), the evaluation of renal function in antenatal period could not be realized because this analysis was not performed in the two university hospital.*

INTRODUCTION

**L66-68: The sentence is a bit unclear. Do the authors mean ‘after’ rather ‘with’ checking of bladder emptying during the exam’ ?**

We thank the reviewer for this remark. Indeed, we mean “after checking” and not “with checking”

*L 66 – 68 : Megacystis in the first trimester of pregnancy is usually defined by a bladder size > 7 mm between 11 and 13^+6^ weeks of gestation (10–12), after checking of bladder emptying during the exam*

**L73-78: The authors should rather justify the interest of the present study by describing the lack of data regarding the prognosis associated with bladder size, so as the etiology depending on the bladder size.**

We agree with the reviewer’s comment. The details have been made

*L 76-78 : However, few authors have specifically evaluated the prognosis associated with bladder size and etiologies (12, 28–30) with, in most cases, limited series.*

**L78-80: The main objective should be rephrased to: ‘…in case of megacystis at first trimester, whether bladder size is associated to unfavorable outcome’**

Changes has been made in the manuscript

*L 78 – 80 : The main objective of this work was to evaluate in case of megacystis of first trimester, whether bladder size is associated to unfavorable outcome.*

**L80-83: I suggest to focus only on etiology based on bladder size (rather than to also aiming to describe the whole series as an objective)**

We agree with the reviewer’s comment. Change has been made in the manuscript

*L 80-81 : The secondary endpoint was description of etiologies and evaluation of whether there was an association between bladder size.*

METHODS

**L95-100 : The authors should justify further the choice of these thresholds.**

As said above, the choice of these thresholds was justified with references. The original article of Kao C. *et al* : *Perinatal outcome and prognostic factors of fetal megacystis diagnosed at 11-14 week’s gestation, Prenat Diagn, 2020 Nov 21. Doi:10.1002/pd.5868*, with a total of 98 cases of first trimester megacystis is very helpful to justify these thresholds.

*L 97 – 99 : For the purpose of this study, three groups were compared according to the work already published (19,29,31): (i) bladder diameter between 7 and 10 mm; (ii) bladder diameter between 11 and 15 mm and (iii) bladder diameter greater than 15 mm.*

**L103-105: It would be very important to identify cases with or without normal renal function among live births if there is a one-year follow-up+++ Dead/Alive is not appropriate for a study on megacystis and renal function is of interest.**

We thank the reviewer for this remark. Indeed, a renal function evaluation is more appropriate than “dead or alive”.

*L 192 – 196 : Of the 12 children with a favorable outcome, 8 (66.7%) had a normal renal function and non-pathological pediatric follow-up. Three children had transitory kidney disease (abnormal transitory renal function) and urinary disorders and needed pediatric urological monitoring. One child had an end-stage kidney disease at birth (abnormal renal function) that required a kidney transplant*

**L109: ‘all patients’ rather than ‘all fetuses’**

Change has been made in the manuscript

*L 108 : All patients underwent detailed sonographic examination twice*

**L116: There was no karyotypes performed in one of the centers???**

Sorry it is a mistake on our part in the manuscript. Karyotypes were performed in the two centers. Corrections have been made in the manuscript

*L 114 : Fetal karyotyping by chorionic villus sampling was systematically offered to the patients after ultrasound diagnosis in the two prenatal diagnosis centers*

**L112-115: Authors should precisely describe the parameters related to the inclusion criteria. How was measured the bladder? Which axis? What if the measurement is different at two different time points during the US examination or between 2 US?**

We thank the reviewer for this comment because he/she’s right.

Change have been made in the manuscript :

*L 94 – 97 : Fetal megacystis was defined as a longitudinal bladder diameter ≥ 7 mm measured from the bladder dome to the bladder neck in the midsagittal plane on an ultrasound performed between 11 and 13^+6^ weeks of gestation and confirmed by an ultrasound specialist. We excluded any diagnosis of megacystis beyond this term.*

RESULTS

**Again, reporting renal function would be really important.**

As said above, we agree with the reviewer’s comment and details were made in the manuscript :

*L 192 – 196 : Of the 12 children with a favorable outcome, 8 (66.7%) had a normal renal function and non-pathological pediatric follow-up. Three children had transitory kidney disease (abnormal transitory renal function) and urinary disorders and needed pediatric urological monitoring. One child had an end-stage kidney disease at birth (abnormal renal function) that required a kidney transplant*

**L176-177: Authors introduce here the evolution of US findings at second US. Is it second trimester US? It would be of interest indeed to have follow-up data but it should be more detailed (including bladder aspect, gestational age,…) in a specific table.**

We thank the reviewer for this comment. The remark is relevant and appropriate. The evolution of US findings corresponds to the second ultrasound examination, one week later :

L 110 – L 112 : All patients underwent detailed sonographic examination twice: a first ultrasound examination and another ultrasound examination one week later for all patients, to assess progression.

The precision was made in the manuscript, line 191 to 211.

Unfortunately we do not have more precise details concerning the ultrasound follow-up. Only data included in the manuscript were reported in the “data table” of this study.

**L184-186: Mixing data from both US is a bit confusing. If inclusion so as tables based on first US, it should be clearly stated in the methods section so as in the results section.**

We thank the reviewer for this remark. We have redefined more clearly the inclusion in methods and results section.

L 112 – 113 : Inclusion has been made when a longitudinal bladder diameter ≥ 7 mm has been found at the first ultrasound.

L 141 – 142 : Seventy-five cases of first-trimester fetal megacystis at the first ultrasound were included in our study population

**L191-216: This section is too long, a bit hard to understand, and not necessary. First information is already provided in Table 3 and above all the article should focus on the outcome based on the bladder size.**

We thank the reviewer for this appropriate comment. This paragraph has been rewritten to be more concise (line 195 to 206)

DISCUSSION

**Discussion is interesting and figure 2 is appropriate.**

We thank the reviewer for this positive statement.

**Reviewer #2: This appears to be a rather large series of a rare fetal condition.
However, megacystis was only transient in a large proportion with good outcomes as expected The authors arbitrarily grouped cases by bladder size. There is no rationale for this and bladder size should therefore be considered a continuous variable There are no information on standardized care pathway or on indications for TOPs The etiologies for LUTO in this series are similar to those reported in previous studies The conclusions are trivial and not substantiated by the data, as explained above.**

Indeed, it is a limit of this work, but as said above, we propose a new analysis in the manuscript by considering the bladder size as a continuous variable. The main objective was to determine two groups, namely to assess the outcome of the pregnancy according to bladder size : one group with favorable outcome, with or without urological problems (n = 12) and one with adverse outcome (n=63). In this analysis, bladder size was considered as a continuous variable. The average bladder size for each group is as follows : 10,2 mm for favorable outcome with a standard deviation of 2,4 and 23,2 mm for adverse outcome with a standard deviation of 10,8, p < 0,001.

We hope that you find our responses satisfactory and that the manuscript meets Journal of Ultrasound in Medicine publication criteria as it currently stands.

Sincerely,

Dr LESIEUR Emmanuelle
