# Supplementary material for: Megacystis in the first trimester of pregnancy: Prognostic factors and perinatal outcomes
Source: PLoS One. 2021 Sep 7;16(9):e0255890. doi: 10.1371/journal.pone.0255890 (PMC8423287; doi:10.1371/journal.pone.0255890)
Supplement: Attachment — Submitted filename: Response to reviewer.docx [file pone.0255890.s003.docx]

**Emmanuelle LESIEUR, MD**

Department of Gynaecology and Obstetrics,

Gynépole, Hôpital Nord

AMU, Aix- Marseille University

AP-HM

France Adress : Chemin des Bourrely

13015, Marseille, France

Phone Number : +33 4 91 96 48 53

Mail : [emmanuelle.lesieur@ap-hm.fr](mailto:emmanuelle.lesieur@ap-hm.fr) ;

To **Editor in chief**

April 2021

Thank you very much for reviewing again our manuscript “***Megacystis in the first trimester of pregnancy: prognostic factors and perinatal outcomes***”. We also greatly appreciate the editor for his complimentary comment.

*Editor comments :*

*The authors should grouped the prognostic category based in the analysis on bladder size and not in a pre-defined bladder size groups, i.e. to analyze bladder size as a continuous variable and selecting the best cut-off to predict adverse outcome. The mean bladder size was significantly higher in cases with adverse outcome, but it remains unknown which is the best cut-off to predict such adverse outcome.*

Our response :

As recommended by this one, we carried out a new statistical analysis using a ROC curve to determine a “bladder diameter cut – off”, making it possible to determine a diameter for which we can predict the neonatal outcome with a high sensitivity and specificity.

Based on the ROC curve analysis, the optimal “cut-off” bladder diameter was 12,5 mm. Under this cut-off, the probability to have a favorable outcome was 91 %, with sensitivity 83,3 % CI 95 (33,3 – 91,7 %) and specificity was 87,3 % CI 95 (75 – 99,9)

Two groups were then compared : (i) bladder diameter under 12,5 mm; (ii) bladder diameter greater than 12,5 mm. Our population was distributed according to this cut off.

All tables and figures have been changed. A new figure, with the ROC curve was created.

Big changes have been made to the manuscript.

We hope that you find our responses satisfactory and that the manuscript meets Plos One publication criteria as it currently stands.

Sincerely,

Dr LESIEUR Emmanuelle
