# Supplementary material for: Megacystis in the first trimester of pregnancy: Prognostic factors and perinatal outcomes
Source: PLoS One. 2021 Sep 7;16(9):e0255890. doi: 10.1371/journal.pone.0255890 (PMC8423287; doi:10.1371/journal.pone.0255890)
Supplement: Attachment — Submitted filename: Response to reviewer.docx [file pone.0255890.s004.docx]

**Emmanuelle LESIEUR, MD**

Department of Gynaecology and Obstetrics,

Gynépole, Hôpital Nord

AMU, Aix- Marseille University

AP-HM

France Adress : Chemin des Bourrely

13015, Marseille, France

Phone Number : +33 4 91 96 48 53

Mail : [emmanuelle.lesieur@ap-hm.fr](mailto:emmanuelle.lesieur@ap-hm.fr) ;

To **Editor in chief**

May 2021

Thank you very much for reviewing again our manuscript “***Megacystis in the first trimester of pregnancy: prognostic factors and perinatal outcomes***”. We also greatly appreciate the editor for his complimentary comment.

*Journal requirements :*

Please review your reference list to ensure that it is complete and correct :

- *Boissier K, Varlet M-N, Chauleur C, Cochin S, Clemenson A, Varlet F, et al. Diagnostic anténatal de mégavessie au premier trimestre de la grossesse. Étude rétrospective sur six années. Gynécologie Obstétrique Fertil. 2009 Feb 1;37(2):115–24.*

This reference has been modified in the manuscript because it was written in French. This has been modified so that it appears in English :

*Boissier K, Varlet M-N, Chauleur C, Cochin S, Clemenson A, Varlet F, et al. Early fetal megacystis at first trimester : a six-year retrospective study. Gynécologie Obstétrique Fertil. 2009 Feb 1;37(2):115–24.*

All other references are correct and correspond to the manuscript. The reference list is complete.

*Editor comments :*

- **Please rephrase the following sentence in the abstract in order to clarify the meaning : "Pathological examination of the fetus was performed in 52 (82.5%)".**

Change has been made :

**“Fetal autopsy was performed in 52 (82.5%) cases of adverse outcome” (l.51)**

- **Please define "neonatal outcome" in the Abstract.**

We thank the reviewer for this remark, it was indeed a missing element. Neonatal outcome was defined in the abstract :

*“The main study endpoint was neonatal outcome based on bladder size. An adverse outcome was defined by the completion of a medical termination of pregnancy (TOP), by the occurrence of in utero fetal death (IUFD), or a neonatal death (ND). Neonatal survival was considered as a favorable outcome and was defined by a live birth, with or without normal renal function, and with a normal karyotype.” (l45.48)*

- **In the Methods section, neonatal outcome has been defined as a live birth with or without normal renal function. Such outcome should be defined as "neonatal survival"**

We agree with the reviewer’s comment. The precision was made in the manuscript :

*“ In this work, neonatal survival was considered as a favorable outcome and was defined by a live birth, with or without normal renal function, and with a normal karyotype” (l.108-110)*

- **The clinical algorithm should include prediction of neonatal survival as a primary outcome and prediction of renal failure as a secondary outcome**

We thank the reviewer for this remark. Changes have been made on the figure 3.

- **Please clarify the meaning of the following sentence "In the case of TPO; pathological examination of the fetus was suggested". Does it mean fetal autopsy?**

We thank the reviewer for this remark. Indeed, it’s much more precise to say “fetal autopsy”. Change has been made in the manuscript.

*“In the case of TOP or intrauterine death, a fetal autopsy was suggested (vacuum system or vaginal delivery)” . (l.128-129)*

We hope that you find our responses satisfactory and that the manuscript meets Plos One publication criteria as it currently stands.

Sincerely,

Dr LESIEUR Emmanuelle
