# Supplementary material for: Megacystis in the first trimester of pregnancy: Prognostic factors and perinatal outcomes
Source: PLoS One. 2021 Sep 7;16(9):e0255890. doi: 10.1371/journal.pone.0255890 (PMC8423287; doi:10.1371/journal.pone.0255890)
Supplement: Attachment — Submitted filename: Response to reviewer.docx [file pone.0255890.s005.docx]

**Emmanuelle LESIEUR, MD**

Department of Gynaecology and Obstetrics,

Gynépole, Hôpital Nord

AMU, Aix- Marseille University

AP-HM

France Adress : Chemin des Bourrely

13015, Marseille, France

Phone Number : +33 4 91 96 48 53

Mail : [emmanuelle.lesieur@ap-hm.fr](mailto:emmanuelle.lesieur@ap-hm.fr) ;

To **Editor in chief**

June 2021

Thank you very much for reviewing again our manuscript “***Megacystis in the first trimester of pregnancy: prognostic factors and perinatal outcomes***”. We also greatly appreciate the editor for his complimentary comment.

*Journal requirements :*

**Please review your reference list to ensure that it is complete and correct.**

All other references are correct and correspond to the manuscript. The reference list is complete.

*Editor comments :*

- **The manuscript has been improved accordingly. However, I strongly recommend to have a grammar correction by a native English speaker because there are still several typographical errors.**

We agree with the reviewer’s comment. Grammar correction has been made by a native English speaker.

We hope that you find our responses satisfactory and that the manuscript meets Plos One publication criteria as it currently stands.

Sincerely,

Dr LESIEUR Emmanuelle
